# Designing Health Care Provider–Centered Emergency Department Interventions: Participatory Design Study

**DOI:** 10.2196/68891

**Published:** 2025-04-21

**Authors:** Woosuk Seo, Jiaqi Li, Zhan Zhang, Chuxuan Zheng, Hardeep Singh, Kalyan Pasupathy, Prashant Mahajan, Sun Young Park

**Affiliations:** 1 School of Information University of Michigan Ann Arbor, MI United States; 2 Seidenberg School of Computer Science and Information Systems Pace University New York, NY United States; 3 Department of Human Centered Design & Engineering University of Washington Seattle, WA United States; 4 Center for Innovations in Quality, Effectiveness and Safety (IQuESt) Michael E. DeBakey Veterans Affairs Medical Center and Baylor College of Medicine Houston, TX United States; 5 Biomedical and Health Information Sciences University of Illinois Chicago Chicago, IL United States; 6 Department of Emergency Medicine University of Michigan Medical School Ann Arbor, MI United States; 7 School of Information, Stamps School of Art and Design University of Michigan Ann Arbor, MI United States

**Keywords:** emergency departments, participatory design, health care providers, technology, interventions, artificial intelligence

## Abstract

**Background:**

In the emergency department (ED), health care providers face extraordinary pressures in delivering accurate diagnoses and care, often working with fragmented or inaccessible patient histories while managing severe time constraints and constant interruptions. These challenges and pressures may lead to potential errors in the ED diagnostic process and risks to patient safety. With advances in technology, interventions have been developed to support ED providers in such pressured settings. However, these interventions may not align with the current practices of ED providers. To better design ED provider–centered interventions, identifying their needs in the diagnostic process is critical.

**Objective:**

This study aimed to identify ED providers’ needs in the diagnostic process through participatory design sessions and to propose design guidelines for provider‑centered technological interventions that support decision‑making and reduce errors.

**Methods:**

We conducted a participatory design study with ED providers to validate their needs and identify considerations for designing ED provider–centered interventions to improve diagnostic safety. We used 9 technological intervention ideas as storyboards to address the study participants’ needs. We had participants discuss the use cases of each intervention idea to assess their needs during the ED care process and facilitated co-design activities with the participants to improve the technological intervention designs. We audio- and video-recorded the design sessions. We then analyzed session transcripts, field notes, and design sketches. In total, we conducted 6 design sessions with 17 ED frontline providers.

**Results:**

Through design sessions with ED providers, we identified 4 key needs in the diagnostic process: information integration, patient prioritization, ED provider-patient communication, and care coordination. We interpreted them as insights for designing technological interventions for ED patients. Hence, we discussed the design implications for technological interventions in four key areas: (1) enhancing ED provider–ED provider communication, (2) enhancing ED provider-patient communication, (3) optimizing the integration of advanced technology, and (4) unleashing the potential of artificial intelligence tools in the ED to improve diagnosis. This work offers evidence-based technology design suggestions for improving diagnostic processes.

**Conclusions:**

This study provides unique insights for designing technological interventions to support ED diagnostic processes. By inviting ED providers into the design process, we present unique insights into the diagnostic process and design considerations for designing novel technological interventions that meet ED providers’ needs in the diagnostic process.

**International Registered Report Identifier (IRRID):**

RR2-10.2196/55357

## Introduction

### Background

Emergency departments (EDs) are dynamic, challenging, and time-critical medical environments in a hospital. In recent years, the increasing need for emergency care services has led to overcrowding [1-3], resulting in extended wait times for patients, diminished patient satisfaction [4], and suboptimal patient outcomes [5,6]. With the high volume of patients and staff shortages [7], health care providers in the ED face significant challenges in their workflow [3,8]. For example, they often encounter frequent distractions and interruptions from secondary tasks or transitions [9-11], ineffective communication with patients [12-14], communication breakdowns, and information loss during the care process [15-18], all while being overwhelmed by the intensive patient and teamwork information [15,19]. All these challenges and issues can potentially lead to diagnostic errors [20-23].

Given these challenges in the diagnostic process in EDs, previous work has attempted to implement different types of technology to enhance this process. In particular, clinical decision support systems (CDSSs) have been implemented to predict morbidity and mortality [24-27], sepsis [28], and adverse prognosis [29]; improve triage [30-35]; automate clinical documentation [36,37]; and predict hospitalization and admission [38-41]. Although these systems enhance ED providers’ decision-making process, many still face barriers to practical applicability and integration into ED workflows [28,42]. For instance, a previous study found that emergency physicians poorly accepted an evidence-based CDSS for evaluating suspected pulmonary embolism because it increased computer time compared to the original workflow, leading to low adoption rates in the ED [43].

One of the main reasons for the failure to adopt and implement effective technological interventions in ED is the limited understanding of ED providers’ technology needs and examining appropriate approaches for integrating advanced technologies into dynamic ED workflows [44]. A way to understand the needs of ED providers is to involve them in the design process [45]. Participatory design (PD) [45] is a methodology that engages all stakeholders in the design process to create solutions that address their needs. Previous work has suggested that participatory and user-centered design approaches can address these concerns [45,46] as they engage all stakeholders in the early stages of the design process, thereby more effectively and promptly addressing user needs and improving the alignment of new technologies with existing workflows [47]. Examples of adopting PD approaches in ED technology development include collaborating with ED providers to design information displays that support awareness and enhance ED teamwork [48] and redesigning the ED patient health information system [49]. Following this body of work, we used the PD approach in our study to explore potential improvements and technological interventions for the ED diagnostic process, an area previously unexplored using the PD method.

### Objectives

In our study, we explored the following research questions: (1) what are the challenges and needs of frontline providers in the ED diagnostic process? and (2) how should technological interventions be designed to address the specific needs of ED providers? To answer these questions, we conducted interviews and PD sessions, engaging ED providers from different hospitals to identify opportunities for creating user-informed technological interventions and to brainstorm strategies for enhancing the diagnostic process in ED. Our study identified 4 primary areas for enhancing the diagnostic process in ED: information integration, patient prioritization, ED provider-patient communication, and care coordination. The findings also highlighted the key concerns about integrating advanced technologies into dynamic ED workflows. Finally, we conclude the paper by discussing design implications for (1) enhancing ED provider-ED provider care coordination and communication, (2) enhancing ED provider-patient communication, (3) optimizing the integration of advanced technology in the ED, and (4) unleashing the potential of artificial intelligence (AI) tools in the ED to improve diagnosis. This work will enhance ED care by offering evidence-based technology recommendations and establishing ED-specific design principles for improving diagnostic processes and care.

## Methods

### Study Overview

This research is part of a comprehensive initiative examining the perspectives of ED patients and health care providers regarding technological interventions to enhance the ED diagnostic process. The broader project aims to develop design guidelines for interventions that address both stakeholder groups' needs. This paper specifically focused on validating ED providers’ needs in the ED diagnostic process and their views on technological support systems. Through 6 PD sessions involving 17 frontline ED providers (n=9, 53% physicians and n=8, 47% nurses), we collected and analyzed session transcripts, design sketches, and field notes. Our analysis revealed 4 key themes that encompassed patient needs, coping strategies for common challenges, and design recommendations for future technological interventions.

### Design Idea Generation Phase for ED Care Interventions

This PD study is part of a larger research project that aims to study diagnostic errors during the ED care process that involves multiple stakeholders, including patients, informal caregivers, nurses, and physicians. Before our PD study with ED providers, the research team interviewed 17 frontline ED providers (n=6, 35% physicians and n=11, 65% nurses) to better understand their experiences and challenges during the ED diagnostic processes. From the interviews, we identified difficulties, emerging patterns of complaints, and general levels of satisfaction with different aspects of the care process (Table 1).

**Table 1:** List of identified problem categories from our previous study’s [50] emergency department (ED) health care provider interview data.

Roles	Problems
Nurses	Missed patient reassessments due to demanding clinical workload. Limitations in tracking patient reassessment timing and clinical status changes.
Physicians	Clinicians’ high levels of stress can impede decision-making and focus on ED work. Physicians’ high cognitive load may interfere with their ED diagnosis work. Lack of decision support tools that aid in diagnostic decision-making for increased accuracy. Difficulty for ED providers to access scattered patient history in a concise and easy-to-read format.
Both nurses and physicians	Insufficient communication between physicians and nurses about orders and next steps in patient care and a lack of EHR^a^ support for such communication. Insufficient communication between physicians and nurses about the patient’s diagnosis and no established opportunity to discuss diagnoses before discharge. Lack of notification and information about incoming patients with critical care needs. Acuity level differs between nurses and physicians. Physicians sometimes have to reassign acuity levels mentally.

^a^EHR: electronic health record.

In addition to the known problems, such as ineffective care team communication, ED providers faced challenges in patient assessment and prioritization before triage, decision-making for patient disposition, and high cognitive load among ED providers. On the basis of the findings, the research team brainstormed numerous design ideas for each problem category, focusing on frontline nurses and physicians. We then merged the design ideas based on feasibility and usefulness. Finally, we narrowed the list and finalized the 9 most effective intervention ideas (Table 2). Each intervention idea aimed to address at least 1 problem category.

**Figure 1:**
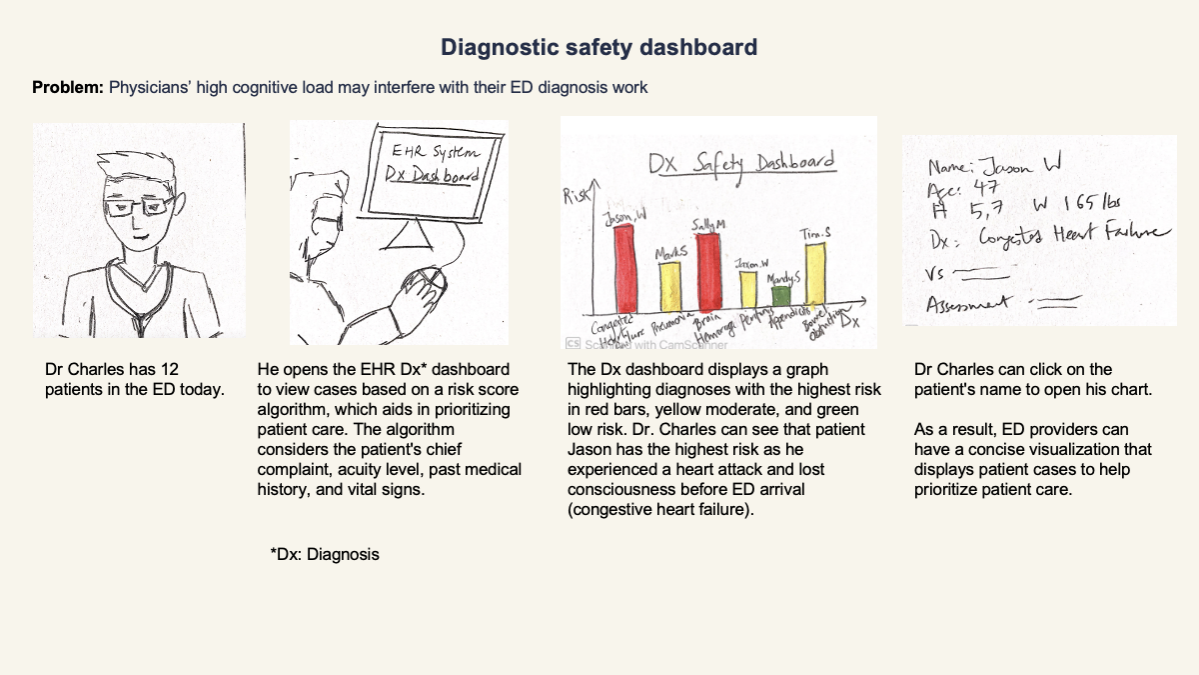
A sample storyboard illustrating how an interactive dashboard for patient risk scores can support the diagnosis process for emergency department (ED) health care providers. EHR: electronic health record.

On the basis of the insights from our interview study, we generated many technological intervention ideas by conceptualizing potential technological interventions that could address the challenges identified in the ED diagnostic process. In addition, we conducted an extensive literature review to understand the status quo, such as the types of technological interventions that had been explored in previous studies and the ED providers’ perspectives on adopting technologies in their workflow (eg, concerns and considerations). These efforts led to the initial generation of >80 intervention ideas, which were then iteratively and collaboratively refined by 3 research team members (WS, JL, and CZ) and 2 medical domain experts. This refinement process resulted in the development of 9 ED provider-facing intervention ideas (Table 2). These interventions were subsequently used to inform and guide participant discussions during PD sessions, which we described in the PD Session Procedure section. To visually illustrate each intervention, we created storyboards for the intervention ideas. A storyboard describes an example of an ED provider facing one or more challenges mentioned above and using the intervention to mitigate the problem (see Figure 1 for a sample storyboard).

**Table 2:** Technology interventions produced in this study along with questions for participant discussion. Participants were asked to rank them based on their preferences and feasibility.

Technology intervention	Description	Sample of lead question used for the discussion of patient need
TI1: machine learning technology for diagnosis support	This intervention introduces an AI^a^-driven tool designed to assist ED^b^ health care providers in diagnosing patients more accurately and efficiently. The system operates by analyzing a combination of patient symptoms, health history, and real-time data, such as nurses’ notes and laboratory results.	“Do you wish you would have more time to spend on complex diagnoses?”
TI2: diagnostic safety dashboard	This intervention introduces a diagnostic safety dashboard designed to assist ED physicians in prioritizing patient care by visualizing risk levels in a clear, color-coded format.	“Do you find it difficult to prioritize patient care mentally based on patient conditions and risk factors?”
TI3: predischarge team huddle	This intervention idea evaluates patient charts for high-risk diagnostic errors before discharge (eg, general diagnosis and other criteria). If flagged, it prompts the care team to initiate a huddle, during which ED providers can review a summary of patient information and assign diagnoses together. This collaborative process ensures alignment on the final diagnosis, reducing the likelihood of errors and improving patient safety.	“Do you feel that more communication is needed between members of the care team about the patient’s diagnosis?”
TI4: visual timeline of the patient’s ED visit	This intervention enhances physician-nurse communication and EHR^c^ support by visualizing the patient’s visit timeline. Nurses can easily access physicians’ orders, notes, and the next steps for patient care, along with key information, such as check-in details, vitals, PERC^d^ scores, and CT^e^ scan results, all in timeline format. This information is accessible both at the nurses’ station and at the patient’s bedside on screen, ensuring that everyone stays informed about orders and the next steps.	“Do you struggle to stay up to date on orders and decisions being made for a patient?”
TI5: patient history aggregator	This intervention addresses the challenge of accessing medical records for new patients with complex medical histories. The PHA^f^ integrates a fingerprint scanner to ensure patient safety and accurately match the patient to their health records from previous hospitals. This allows ED providers to quickly access crucial information about the patient’s heart disease and other past conditions, ensuring informed and efficient diagnostic decisions.	“Do you find it difficult to review a patient’s history in a summarized format, especially when it’s scattered across multiple EHR systems?”
TI6: EHR-prompted diagnostic pause	This intervention features an EHR-integrated diagnostic pause to reduce errors in complex cases. When a patient presents with symptoms such as abdominal pain and fever, the initial ED provider records their diagnosis. For complex cases, the EHR alerts the next ED provider to independently evaluate the patient before viewing the first diagnosis, which remains hidden until completion. If diagnoses differ, the system recommends team discussion. The alert links to relevant patient information for easy reevaluation.	“Is there a higher risk of diagnostic error for more complex cases?”
TI7: real-time EMS^g^ information via smart glasses	This intervention addresses the challenge of unexpected critical patient arrivals by enhancing communication between EMS and the ED care team. Using smart glasses, EMTs^h^ can initiate a real-time video call with the ED care team, including physicians and nurses. This allows the care team to view the patient’s condition from the EMT’s perspective and receive critical information en route to the hospital.	“Do you wish you had more information about when patients are being transferred to the ED, and what sort of preparation is needed?”
TI8: reassessment reminder door flag	This intervention introduces a reassessment reminder system to ensure timely and efficient monitoring of patient conditions. When a time-sensitive reassessment, such as checking vital signs or evaluating treatment effectiveness, is due, a display monitor outside the patient’s room flashes a green light as a visual alert. The monitor also displays key details, such as the medication administered and the task that needs to be completed. Nurses can see the flashing light from the nurses’ station or other areas, prompting them to respond.	“Are you tired of relying on your memory and constantly looking at the clock in order to complete nursing tasks such as patient reassessments every hour or so?”
TI9: stress management tracking	This intervention introduces a stress management tracking system to monitor and support the well-being of health care providers during demanding shifts. The system includes a wearable device that tracks indicators, such as heart rate and sleep cycles. When abnormal stress levels or poor sleep patterns are detected, the device notifies the wearer with an alert, accompanied by a message suggesting a break or rest. This ensures that ED providers’ health is prioritized, reducing stress and fatigue while improving focus, performance, and overall cognitive function during their shifts.	“Have you ever felt so stressed that you were not able to focus or found it difficult to make decisions?”

^a^AI: artificial intelligence.

^b^ED: emergency department.

^c^EHR: electronic health record.

^d^PERC: pulmonary embolism rule-out criteria.

^e^CT: computed tomography.

^f^PHA: patient history aggregator.

^g^EMS: emergency medical service.

^h^EMT: emergency medical technicians.

### Participant Recruitment

Participants in the study were recruited from 2 large health care organizations in the United States. Organization A is a university-affiliated medical institution in the Midwest, and organization B is a renowned, comprehensive medical center also located in the Midwest. Eligible participants included ED physicians and nurses who had worked in the ED setting for at least 1 year. A study coordinator contacted potential participants via email or in person on the day of their shift. Once eligible ED providers expressed interest in participating, we obtained electronic informed consent by emailing the form to them before each session. If participants did not complete the form in time, we provided a paper copy at the start of the session for in-person completion. All PD sessions were audio recorded and transcribed for data analysis. In total, 17 clinicians (n=9, 53% physicians and n=8, 47% nurses) participated in 6 PD sessions.

### PD Session Procedure

We conducted a total of 6 PD sessions. Each session consisted of 3 to 4 participants with the same or similar roles (eg, nurses or physicians) to mitigate the potential influence of power dynamics on the expression of opinions [51,52]. The participants’ characteristics varied in experience, from novices to experts with >30 years of experience in the field, as detailed in Table 3. The sessions were conducted either in person or online via Zoom (Zoom Communications, Inc), depending on the participants’ preferences and availability. We provided colored pens, paper, and sticky notes for in-person sessions, while for virtual sessions, we used slide presentations and a Miro board (Miro, Inc) as interactive whiteboards for the participants’ design collaboration. In addition, 2 to 3 researchers were present at each session, taking detailed notes.

Each PD session lasted up to 120 minutes and comprised 3 major parts: intervention idea discussion, individual design, and group design. Specifically, the first part focused on the presentation and discussion of the 9 intervention ideas developed by the researchers. After presenting each idea, we used leading questions to assess the perspectives of ED providers (eg, needs, opinions, perceptions, and expectations) for each intervention idea and allowed them to rank the intervention ideas (Table 2). In the individual design activity, participants selected 1 or 2 intervention ideas that most resonated with their needs based on their personal perspectives and professional experiences. After choosing their preferred intervention ideas, they were asked to further improve the design by modifying or adding new elements and features or to design their own new intervention. For the group design activity, participants were asked to select a top intervention idea as a group and work together to synthesize their individual design ideas and develop a final design solution (see Multimedia Appendix 1 for sample group designs). Participants engaged in discussions on the salient features, pros and cons, and concerns associated with their final group design. Each session was concluded by eliciting participant feedback to improve future sessions.

**Table 3:** Demographic information of study participants for each design session.

Session	Format	Affiliation	Occupation	Participant ID	Experience (y)
Session 1	Remote	Organization A	Physicians	Participant 1	20
Session 1	Remote	Organization A	Physicians	Participant 2	5
Session 1	Remote	Organization A	Physicians	Participant 3	16
Session 2	In person	Organization A	Physicians	Participant 4	19
Session 2	In person	Organization A	Physicians	Participant 5	31
Session 2	In person	Organization A	Physicians	Participant 6	2
Session 3	In person	Organization A	Nurses	Participant 7	7
Session 3	In person	Organization A	Nurses	Participant 8	15
Session 4	Remote	Organization B	Physicians	Participant 9	15
Session 4	Remote	Organization B	Physicians	Participant 10	26
Session 4	Remote	Organization B	Physicians	Participant 11	15
Session 5	Remote	Organization B	Nurses	Participant 12	8
Session 5	Remote	Organization B	Nurses	Participant 13	9
Session 5	Remote	Organization B	Nurses	Participant 14	20
Session 6	Remote	Organization B	Nurses	Participant 15	30
Session 6	Remote	Organization B	Nurses	Participant 16	3
Session 6	Remote	Organization B	Nurses	Participant 17	2

For the in-person sessions, we captured audio recordings and took photographs of the participants’ sketches and designs. For virtual sessions, video recordings were made via Zoom, and participants’ digital creations were also recorded for further analysis. In addition to the recordings, session facilitators took field notes throughout the sessions for data analysis.

### Data Analysis

We used transcripts as the primary data source, and all other collected data (eg, observation notes) were used as supplementary sources. We also incorporated the co-design activity sketches as supplementary data to validate what participants discussed during the session and clarify participants’ design suggestions.

After organizing the collected data, 2 researchers open-coded the transcripts of the first 3 PD sessions. The researchers also analyzed content from participants’ sketches and design illustrations, converting these visual elements into textual descriptions to facilitate thorough examination. Each sketch or digital artifact was meticulously analyzed to extract key features, such as design elements, functional aspects, and underlying needs. The codes were compiled into a growing codebook organized by their corresponding intervention or put into a general category. On the basis of this evolving codebook, the remaining transcripts were then coded. If any new codes were discovered, they were either added to the codebook as a new code or incorporated into the definition of an existing, similar code.

We then used affinity diagramming [53] to identify the emerging themes from the codes. We used the Miro board for affinity diagramming. Similar codes were first grouped together into subthemes specific to each intervention. Then, a team of researchers engaged in a series of discussions to build larger, emerging themes that spanned across the different interventions. For example, a larger theme of “communication among the care team members” contained various subthemes, such as “piece together information about a patient and keep every care team member on the same page.” Codes that were ambiguous or needed further support were refined by referencing participant quotes from transcripts and participants’ sketches from PD sessions. After multiple group discussions, we identified 4 themes that described ED providers’ challenges in the diagnostic process.

### Ethical Considerations

We complied with the following ethical considerations. First, we obtained consent from all participants before the study sessions, which were approved by the multisite institutional review board at the University of Michigan (HUM00156261). Participants were informed that their participation was voluntary, the sessions would be recorded, the collected information would be deidentified and protected in secured storage, and they would be compensated with US $100 for their participation. Second, we ensured that the collected transcript data were deidentified by replacing participants’ names with pseudonyms, and that their faces in photos or videos were blurred. All collected data are stored in secure storage that requires the university accounts for access. Third, all the researchers completed research compliance training, such as the Collaborative Institutional Training Initiative or the Program for the Education and Evaluation in Responsible Conduct of Research, to become trained on best practices and ethical considerations for interacting with ED providers and patients.

## Results

### Overview

On the basis of the analysis of collected data, we identified top intervention ideas that participants preferred, 4 key areas in the ED diagnostic process requiring technological support, and major concerns regarding the integration of advanced technologies into dynamic ED workflows.

Throughout all sessions, participants collectively favored the following interventions to enhance the existing ED diagnosis process (Table 2): TI1 (machine learning technology for diagnosis support), TI2 (diagnostic safety dashboard), TI3 (predischarge team huddle), and TI4 (visual timeline of the patient’s ED visit). Participants believed that these intervention ideas could improve crucial aspects of the ED diagnosis process. Specifically, TI1 could broaden access to comprehensive patient information and reduce potential errors. TI2 might simultaneously manage multiple patient cases, optimize prioritization, and rapidly identify high-risk patients. TI3 could address communication and understanding gaps between physicians and nurses regarding diagnoses, while TI4 aims to bridge the communication divide and keep patients informed about their care progress.

Furthermore, we reported the key areas of improvement in the diagnostic process, which indicated the challenges faced by ED providers in the diagnostic process. We present 4 prominent aspects extensively discussed among participants in the subsequent sections.

### Effective Integration of Patient Information for Timely and Accurate Diagnosis

Participants often faced challenges with incomplete, hard-to-find, or misleading patient information, which can inadvertently lead the medical team down an incorrect diagnostic pathway. Incompleteness can result from prehospital care providers failing to relay all necessary information at handoff or integration issues among health care information systems. Assessing relevant information is problematic when stored using varied and repetitive terminologies or buried in multilevel systems. Misleading information typically occurs with objective data, such as vital signs. For example, external stress or the use of an incorrectly sized cuff can result in elevated heart rates or blood pressure readings, potentially skewing risk assessments.

To access comprehensive patient information effectively, participants mentioned that both historical information (eg, past medical history, long-term medications, patients’ care preferences, and social determinants) and ad hoc information (eg, laboratory test results, vital signs, and clinical observation notes) should be integrated to make an accurate diagnosis. For instance, Participant 16 suggested how TI5 (patient history aggregator) could be integrated with existing care systems to help ED providers in the diagnosis process as follows:

If we could have that [History Aggregator] page already incorporate all the other systems where this patient has received care, I see that as helpful. It’s kind of like, in my mind, redefining the chart review tab in a more encompassing form.

Participant 16

Some participants also highlighted the dynamic changes in physiological values when redesigning TI1 (machine learning technology for diagnosis support). To maintain accurate risk assessments, it is critical to continuously update data reflecting the dynamic changes in patient conditions, especially for those patients with critical illness whose health status can rapidly evolve. This suggestion showed participants’ desire to receive updates about the patient’s status for better management, as many patients are coming in and out of the ED.

Some participants suggested that medical information should be displayed in a simple, clear, and efficient manner, such as streamlining all necessary information onto 1 page to avoid spending time searching and organizing it piece by piece. Participant 5 highlighted the importance of information presentation for TI4 (visual timeline of the patient’s ED visit) as follows:

It’s information that we just want to be displayed in a more graphic, simpler form where it’s not so busy. You open it up, it’s one snapshot, and you click on which part of it you want to do now...It’s just organized in a better way, in a simpler way.

Participant 5

Participant 5 envisioned that this approach would enable health care providers to efficiently focus on critical details, such as a patient’s historical data, directly relevant to their chief complaint.

### Optimizing Patient Prioritization to Improve the Triage and Care Process

Another important area to improve was related to prioritizing patients. The current triage process may lead to the improper prioritization of patients with critical needs. Triage in the ED can be influenced by many factors, including triage nurses’ clinical knowledge and judgment, the clarity with which patients describe their symptoms, and the availability of patient records or medical histories. In addition, the Emergency Severity Index (ESI) classification criteria, which classify and prioritize patients based on the severity of their disease and the estimated number of resources needed for their care, can fail to account for dynamic changes in a patient’s condition. For example, there was no adaptation of the ESI score despite clear signs that the patient had become more critically ill. For these reasons, there is a risk of patients being misclassified and inappropriately prioritized, which may result in those with severe conditions facing longer wait times.

To address those issues in the triage process, our participants expected the AI-driven prioritization system to analyze various types of information, including contextual (patient’s relevant medical history and symptoms), situational (dynamic changes in patients’ symptoms and length of waiting time), and human factors (clinician’s knowledge and experience). This ensures timely attention to critical cases and improves ESI’s shortcomings. For instance, when discussing idea TI2 (diagnostic safety dashboard), participants stated that the algorithm could consider different and comprehensive factors to identify higher-risk individuals, such as those with a history of asthma or cardiac problems. A participant noted as follows:

The algorithm looks at the chief complaint, acuity level, and past medical history. I think that helps as well. For example, if it’s a younger person with a syncopal episode and you find out they have a previous cardiac history, that would make them a higher risk than, say, a 30-year-old who passed out for another reason. I like how this algorithm considers the whole patient and takes that into account.

Participant 12

In addition, many participants valued the explicit representation of different patient prioritizations through various colors (eg, red, yellow, and green) and symbols (eg, an alarm bell and a smiley face) because it could enable ED providers to identify which patients require immediate attention visually quickly. For instance, Participant 8 preferred color coding of data visualization for immediate understanding of priority as follows:

I like the color because then they could look at that and say that they’re at high risk. If they’re above five risk scores then they are red or something, and then they’re like, “Oh, I have all these priority 2s as Joe is sicker.”

Participant 8

### Enhancing Patient Engagement in the Diagnostic Process

Our participants mentioned that it could be challenging for ED providers to receive timely diagnostic feedback and updates on changing symptoms from patients due to time constraints and heavy workloads in the ED. ED providers often rely on brief interactions or 1-way communications to gather information, which may lead to an incomplete understanding of patients’ evolving symptoms. In addition, patients frequently feel anxious and frustrated due to the lack of ongoing updates about their condition, which can lead to increased complaints and misunderstandings.

Many participants agreed that informing patients about the status and progression of their care could help manage their expectations and alleviate anxiety. They expected patients to feel reassured that ED providers would still take care of them when patients understood the next steps in the diagnosis process. Participant 2 stated as follows when discussing idea TI4 (visual timeline of the patient’s ED visit):

The patient can visualize what’s been done and what they’re waiting for. It sort of wards off that sentiment of, “I went to the ER, and they didn’t do anything,” and also gives them an idea of what the next step is.

PD-2

By sharing the ED process steps, participants anticipated more patient engagement and having more time to focus on effective patient communication.

Participants mentioned they wanted easy-to-use tools to help receive more accurate, timely updates directly from patients to aid the care process. Nurses’ work could be expedited by having patients more actively involved in their care and providing information. For example, participants suggested that the idea TI8 (reassessment reminder door flag) could include a feature allowing patients to undertake a self-reassessment of their current condition and medication reactions and relay their results and updates to nurses, potentially via an automated prompt and chat system on a tablet or phone. If a nurse records the administration of fentanyl at noon, the system might automatically inquire about the patient’s pain levels 20 minutes later. Then, patients could interact with the nurse, who can gauge the treatment’s effectiveness, as noted by a participant:

I wonder if there’s a way for the patient to enter a self-reassessment like they have their own little iPad and can share how they’re doing, whether the pain medication helped their pain rating...it gives the patient the opportunity to provide feedback.

Participant 9

Our participants also mentioned that engaging patients in diagnostic discussions could be crucial to reducing potential diagnostic errors. This could allow ED providers to elucidate the logic behind a diagnosis, articulate how tailored care plans were developed for individual patients, and seek the patient’s perspective regarding the proposed plan. In addition to the illustrated functionality of increasing care team communications to reduce diagnosis errors from ideas TI3 (predischarge team huddle) and TI6 (electronic health record (EHR)–prompted diagnostic pause), participants suggested empowering patients to contribute supplementary information and express different viewpoints that might not have been considered, to reduce diagnosis errors. For instance, Participant 3 emphasized that patients should have the opportunity to share their perspectives on ED providers’ reasoning as a way to promote patient engagement in diagnostic discussions as follows:

If the patients heard the diagnostic reasoning, you [clinicians] would be giving them an opportunity to correct you [clinicians].

Participant 3

### Care Coordination Among the ED Team Members

Through the sessions, we identified that the ED care team faced 2 main communication issues. First, each member of the ED care team has only partial information, leading to a disjointed and fragmented understanding of the patient’s case, primarily due to interactions at different stages of care. Patients move from 1 ED provider to another, and no one fully knows all the details of the patient’s condition. Furthermore, its interpretation can differ even when information is consistently shared among team members. This variation in understanding can be attributed to individual backgrounds, areas of expertise, or perspectives. Second, physician-to-nurse communication could sometimes become ineffective, especially regarding discharge and diagnostic reasoning information. For example, physicians sometimes fail to communicate the rationale behind diagnoses, orders, or care plans to nurses. This issue can impede nurses’ understanding of physician decisions and their ability to respond accurately to patient queries. Furthermore, participants acknowledged that this communication issue may be derived from both sides, as noted by participant 7:

[Physician-nurse communication] is a mutual problem, it’s not a one-sided issue.

Participant 7

All participants agreed on the importance of maintaining a consistent and uniform understanding among care team members, especially for complex patient cases. Engaging more care team members in discussions could be advantageous. When discussing idea TI3 (predischarge team huddle), participants noted that it would offer an excellent opportunity for comprehensive information gathering from different team members and roles to minimize potential errors. A participant highlighted as follows:

You would get everyone on the same page and give the patient a chance to ask questions. At the last minute, if someone asks, “Well, what about this, or what about that?” everyone is on the same page, which I like.

Participant 2

Most physicians and all the nurses mentioned the need for physicians to communicate more detailed information with nurses, including the reasoning behind diagnoses, orders, and care plans, to enable nurses to understand the overall situation comprehensively. When discussing idea TI4 (visual timeline of the patient’s ED visit), physicians believed that this technological intervention could be instrumental in keeping nurses informed about care plans and the rationale behind physicians’ orders in a timely manner. This enhanced transparent communication could empower nurses to answer patients’ queries more effectively, reducing the need for ambiguous or noncommittal responses. Nurses, in particular, advocated this because they wished to better explain physicians’ orders and diagnoses to patients, as one participant noted:

Sometimes I go into the patient room and say, “I think they [the physicians] ordered this because they’re probably looking to see if you [the patient] have this issue.” It would be nice to say confidently, they ordered this because they’re looking to see that you have this issue.

Participant 8

Among our participants, some care team members might hesitate to point out potential errors, from junior members to senior members or nurses to physicians. For example, nurses might be reluctant to bring up potential diagnostic errors to physicians due to a perception that physicians, being more knowledgeable and experienced in diagnosis, are responsible for treatment decisions. However, the participants believed that some technologies, acting as second opinions, might help address the power dynamic issue. For example, when discussing the idea TI1 (machine learning technology for diagnosis support), nurses expressed that the list of diagnoses generated by machine learning tools could empower them to engage in discussions with physicians regarding potential misdiagnoses significantly when the physician’s conclusion deviates from the algorithm’s suggestion, as noted by a participant:

It [the machine learning technology tool] also brings up good discussion points with the physician...why are we pursuing this route...or why we’re deviating from a particular diagnosis.

Participant 14

### Concerns of Integrating Advanced Technologies in ED

#### Overview

Our 9 intervention storyboards described how emerging technologies can potentially be designed to support the ED diagnostic process. While the storyboards were primarily used to validate ED providers’ needs, they were also used for discussions about potential issues of integrating such technological interventions. This section presents participants’ concerns regarding integrating advanced technologies in ED, including the safe use of AI, privacy, and additional cognitive and operational workload.

#### Safe Use of AI-Empowered CDSSs

With the growing application of AI techniques in health care, more than half of our intervention ideas were inspired by AI-empowered tools. Thus, many participants expressed concerns about the AI’s potential inability to consider different care contexts and individual nuances of each patient case in analyzing and presenting various information. This concern was mainly raised during group design activities on several AI-driven interventions, such as TI1 (machine learning technology for diagnosis support), TI2 (diagnostic safety dashboard), and TI4 (visual timeline of the patient’s ED visit). Participants particularly mentioned that the same test result can be interpreted differently based on the context. For instance, a particular urine analysis result can indicate different health conditions depending on whether the patient has a positive or negative ultrasound result, as noted by a participant:

The same urine analysis result means something totally different if you tell me that the patient has an ultrasound that’s clearly positive than if they have an ultrasound that’s clearly negative, and it can have the exact same result. And that’s the problem. It comes in context.

Participant 4

Participants suggested that AI systems should account for various factors and contextual cues, such as interpreting test results with other diagnostic information and medical history. However, it may not fully match human ED providers’ ability to perceive subtle cues and insights, which could be beyond the scope of computers or AI systems. In addition, participants suggested that the AI systems should apply different weights to different diagnostic factors (eg, laboratory results and medical history) because certain factors weigh more than others, or the system should adjust weights based on dynamic emergency severities when calculating risk scores.

In addition, participants underscored the imperative need for rigorous testing and validation before implementing any AI tool in their existing work practice. They believed that an AI tool must prove its reliability and efficacy to gain the trust of medical providers, as noted by a participant:

Critical for it to be, the tool really has got to be tested and robust before it’s implemented. Otherwise, no one’s going to entertain the idea of adopting it because it’s just going to create extra work without perceived value.

Participant 10

Participants also expressed concerns that an overreliance on technology could potentially lead to a degradation of human clinical skills and emphasized the balance between such emergent technology use and human skills. For instance, when discussing the idea TI1 (machine learning technology for diagnosis support), there was a worry that ED providers might depend too heavily on technology and neglect their assessments. Participant 13 expressed her concern about potential overreliance on AI tools as follows:

The only concern I can think of right now is...becoming too reliant on AI...If any residents or doctors would start to rely on it too much...you’d hope not, you’d hope they do their own assessments.

Participant 13

As a potential solution to mitigate such overreliance, some participants suggested that they would like to incorporate AI-powered CDSSs into their on-the-job training. This integration could allow ED providers to learn from these tools and understand how to collaborate with them effectively without overly relying on AI outputs while maintaining their clinical assessment skills.

#### Privacy Concerns Toward Emerging Technologies

Participants repeatedly mentioned privacy concerns related to new advanced technologies. They raised issues about potential violations of the Health Insurance Portability and Accountability Act and patient confidentiality, emphasizing the need to ensure adherence to patient privacy laws and regulations. For example, TI5 (patient history aggregator) allows easy access to patient history by scanning patients’ fingerprints. Many participants expressed concerns about collecting sensitive biometric data (ie, fingerprints) from their patients. Participant 16 brought up the issue of data storage that may lead to a privacy breach, as follows:

If it is based on fingerprint, where is the information being housed? Is it a cloud database? That is universal? Is it an extension of the electronic health record? Where does it belong? I think it will be a question.

Participant 16

Participants’ such concerns about privacy highlight the importance of transparent communication regarding data storage practices, robust security measures, and clear protocols for accessing sensitive patient information to foster trust in emerging health technologies.

#### Extra Workload and Burden to Make Technology Fit Into Their Existing Workflow

Participants raised concerns about the potential for certain technological implementations to interfere with the established ED workflow, possibly creating extra waiting time for patients and adding extra cognitive and operational workload for ED providers. One notable instance occurred during the discussion of the idea TI3 (predischarge team huddle). Some participants expressed that integrating such a system could inadvertently delay or create a backlog in the patient discharge process. Given the current ED workflow, it could be challenging to convene all care team members due to their individual duties and potential busyness. A participant explained this concern by stating as follows:

When you have a patient who is itching to get out the door [being discharged], and you say, “while we’re waiting for this person to show up or we need this other team member [to discuss again],” it can create a lot of delay and backlog if now you have to round to discharge everybody.

Participant 2

Another example arose during the discussion on the TI6 (EHR-prompted diagnostic pause), which highlighted concerns that its implementation could disrupt the typical ED workflow by introducing a “parallel” evaluation process. In this context, “parallel evaluation” means that multiple ED providers might independently diagnose the patient, leading to repetitive and time-consuming efforts. This could result in a prolonged diagnostic process and potentially delay treatment, as noted by a participant:

I think the scenario would require a change in our current flow, where it’s sequential in terms of evaluations that are done of a patient...To one where there’s a parallel evaluation, which I think becomes very inefficient and for a patient, can be quite redundant as they have to repeat the story multiple times.

Participant 10

## Discussion

### Design Implications

#### Overview

Our findings show that the ED diagnostic process can be improved into 4 main areas: information integration, patient prioritization, ED provider-patient communication, and care coordination. We also summarized the key concerns of ED providers in integrating advanced technologies in the ED, including the safe use of AI, privacy, and the additional cognitive and operational workload. Drawing from these findings, we discuss potential design implications of future ED-centered intervention tools for improving the ED diagnosis process.

#### Creating Standardized Communication Procedures

We observed that there is significant demand for improving communication between ED providers (the idea TI3 [predischarge team huddle] was ranked third by participants, and TI4 [visual timeline of patient’s ED visit] was ranked fourth by participants). Information sharing between ED physicians and nurses is often inadequate, limiting nurses’ comprehensive understanding of patient conditions. In addition, varying levels of understanding about the patient exist among different stages in the care team, further complicating the communication process. These findings highlight the importance of establishing standardized communication procedures to ensure a unified understanding of patient conditions among team members. We suggest creating more standardized communication procedures in future technical tool designs. Building on previous insights from the design of digital checklists for trauma resuscitation [54], we recommend implementing an automated checklist system that includes critical patient information, such as diagnosis, medication orders, and care milestones. This checklist should be integrated with a timeline that automatically captures details of when each piece of information was added and when action needs to be taken, ensuring that all team members are aware of current in-progress and upcoming responsibilities. in addition, structured communication features, such as mandatory read-backs of critical information, regular briefings at shift changes, and digital alerts for key updates, can further enhance understanding and collaboration. Such tools are particularly helpful for nurses, who often coordinate various aspects of patient care and benefit from clear, concise, and timely information to make informed decisions and provide the best possible care. This potential application direction also echoes the previous study on the necessity of applying new technological communication interventions to simplify communication among various roles within the ED [55].

#### Incorporating Technology-Based Second Opinions

We identified power dynamics as a critical issue in the diagnostic process. Such skewed interactions are prevalent between junior and senior members, as well as between nurses and physicians. Hesitation in pointing out potential errors and reluctance to accept feedback is not uncommon, and it could have severe consequences (eg, increased incidence of medical errors and significant patient safety risks) in fast-paced and critical medical fields such as EDs [51,52]. Although more collaborative approaches, such as education and mentoring, have been proposed to address the issue of power dynamics [56], we found that this issue has not yet been fully resolved. Notably, our findings indicated the potential of technological interventions in mitigating these power dynamics. For instance, the timeline in idea TI4 (visual timeline of the patient’s ED visit) could allow nurses to understand the rationale behind care plans, enhancing their ability to communicate with physicians actively, thus promoting equal and open communication within the team. Furthermore, as mentioned in idea TI1 (machine learning technology for diagnosis support), second opinions generated using machine learning tools could boost the confidence of junior physicians or nurses in discussing potential misdiagnoses with senior members or physicians in the care team. This echoes and expands on past research [57] about the second opinion in physician-patient relationships, demonstrating it to be an effective way to promote open, positive communication and to enhance satisfaction with clinical decisions. By providing second opinions and facilitating information sharing, technological interventions could help overcome the barriers in the traditional power structure of medical roles, potentially improving overall care quality and patient safety. Future work could involve designing and implementing such information tools in ED settings to evaluate potential impacts on the ED diagnostic process. For example, in the new ED medical display system, each team member could autonomously select CDSS reports that automatically highlight decision details differing from the primary physician’s, with junior members and nurses also accessing these for reference and discussion as a second opinion.

#### Empowering Patients With Information Transparency

We found a demand for improved communication between ED providers and patients through timely information sharing, transparency with patients, and patient empowerment. Previous studies show that insufficient communication between patients and ED providers in the ED leads to dissatisfaction with their care and potential diagnosis errors [12,58]. Factors contributing to their communication issues include patients’ and ED providers’ conflicting perceptions of the ED, a lack of patient information, and physicians’ diagnostic time pressures [12,58,59]. Our study further reveals the specific manifestations of insufficient communication in the ED between patients and ED providers: (1) patients are not fully informed about the treatment process; (2) lack of opportunities for patients to provide feedback and update their conditions to ED providers actively; and (3) absence of processes for joint decision-making and care planning between ED providers and patients, as well as effectively involving patients in diagnostic discussions. These communication deficiencies can lead to patient anxiety and an increased risk of misdiagnosis. Therefore, we suggest that future technological interventions should focus on enhancing timely information sharing and transparency with patients and significantly empowering patients’ agency. This involves promoting active patient participation in communication, even in joint decision-making and care planning. Previous research [36] discussed a digital ED waiting room history-taking tool that uses reasoning engine functions to gather patient information. It has been proven to improve communication and understanding between patients and ED providers. Building on this foundation, we suggest developing a mobile app that allows patients to promptly report changes in their symptoms or express concerns using real-time data reflecting their current condition and personal feelings during their ED visits. The app would also integrate features that encourage patient involvement in diagnostic discussions. Such features could include a symptom timestamp for patients to log details of their symptoms as they occur and a communication chat window that allows for direct dialogue with ED providers.

#### Optimizing Advanced Technology Integration in the ED

Our research highlighted specific considerations for integrating advanced technologies into the ED context: minimizing additional workload and cognitive burdens for ED providers and using high-fidelity simulations. Unlike other general clinical practices where there is more time to adapt and use new technology, our findings indicated that ED providers in the fast-paced ED environment may not have the time to try out, thoroughly examine, and use these technologies. Furthermore, they were reluctant to change their existing workflows to accommodate new technologies, fearing the risk of additional workload. During the design of information displays, our ED providers preferred concise and practical interfaces, capturing essential information immediately rather than increasing their cognitive load by requiring them to understand the meaning of every icon and content. In addition, participants considered idea TI6 (EHR-prompted diagnostic pause) ineffective and redundant, despite its merit of catching potential diagnostic errors, as it does not fit their existing workflow. Similarly, during the discussion of idea TI3 (predischarge team huddle), participants acknowledged the critical need for care team members to be on the same page regarding information sharing upon patient discharge. However, they still harbored concerns that the intervention would not align well with their current workflow or the realistic situation where patients would not want to stay longer and wait for a reevaluation before their discharge. Therefore, building on previous research [60] that emphasized the need for efficiency in the design of ED technological interventions, we recommend that future designs and implementations of ED technological interventions consider the fast-paced, high-pressure nature of the ED. This involves prioritizing efficiency and focusing on avoiding additional workload and cognitive burdens for each ED provider. For example, AI-driven decision support tools should be overseen by designated care team members who monitor AI-recommended diagnostics and conduct departmental audits and custom improvements to reduce the burden for individual ED providers. Similarly, designing interventions, such as the idea TI3 (predischarge team huddle), should minimize the potential for increasing workload. Instead of prompting the care team to initiate a team huddle to reevaluate, the design should allow members to quickly review summary reports in the EHR to identify potential issues with minimal additional effort.

From a methodological perspective, we also recommend building on existing ED workflows using methods such as “group workshops” similar to PD or “high-fidelity team-based simulations” conducted in clinical scenarios [61,62]. These methods could allow ED team members to experience new technologies and foresee how they can be implemented in their existing work practices while gathering feedback. High-fidelity team-based simulations have been widely used in the ED for effect evaluation and training, such as improving communication among ED provider teams [63] and detecting latent safety threats in critical patients [64], proving very effective. Therefore, we suggest extending this approach to introducing new technologies in the ED to guide the integration of new advanced technologies.

#### Leveraging AI Tools in the ED to Enhance Diagnosis Through Dynamic Patient Data

Our research highlighted a strong demand from our participants to unleash the potential of AI tools in the ED to improve diagnosis further. We found that participants highly valued the top 2 AI-related ideas (TI1 [machine learning technology for diagnosis support] and TI2 [diagnostic safety dashboard]) because of their potential to (1) integrate a broader range of comprehensive patient information and perform multidimensional data analysis and (2) facilitate dynamic prioritization and resource allocation. Although there are multiple existing AI applications in the medical field, many of them lack consideration of multidimensional information and dynamic prioritization [65] and thus do not fully unleash the potential of AI. To best leverage AI tools in future ED settings, we suggest that when designing algorithm development for ED, the input information for these algorithms should be multidimensional and incorporate dynamic patient data. These algorithms should be capable of continuously monitoring and evolving based on real-time patient data and feedback, which can aid in enhancing diagnostic accuracy and dynamically adjusting treatment protocols. For example, when designing AI tools for patient prioritization, it is crucial to implement a system that categorizes patients based on initial information and updates its prioritization as new data becomes available (eg, laboratory results or vital signs). This approach could ensure that resources are allocated promptly to patients with escalating needs.

### Limitations

A few limitations of this study should be noted. First, one of the primary limitations of this study relates to the representativeness of the participant sample. Participants were recruited from only 2 large health care organizations in the Midwest region of the United States. While these organizations serve millions and can offer significant insights, this sample may not fully encapsulate the diversity and range of experiences of ED providers worldwide. There might be differences in health care systems, cultural contexts, and operational protocols in EDs across different countries or regions. Another limitation of this research is inherent in the study design, particularly regarding the PD sessions. Each session was constrained to a duration of around 120 minutes. This limited timeframe could have impacted the depth and extent of interaction among participants, potentially affecting their comfort levels and the quality of their responses. Participants might have required more time to fully engage with the material, express their thoughts, and collaborate effectively, influencing the innovation and creativity aspects of the session’s outcomes. Furthermore, some PD sessions were conducted in a virtual format. While virtual sessions offer flexibility and can include a broader range of participants, they might lack the richness of interaction found in face-to-face settings. Nonverbal cues, ease of spontaneous conversation, and the organic development of collaborative ideas are often diminished in virtual environments. The potential limitations in participant interaction and engagement due to the virtual format of some sessions might have affected the overall effectiveness and output of these PD sessions. Finally, our study is limited by not presenting functional systems but merely asking participants to imagine how the system works. Without seeing or interacting with a system, eliciting comprehensive user feedback could be challenging.

### Conclusions

This work presents opportunities for improving ED provider–informed technological interventions and strategies for enhancing the diagnostic process in EDs. Our study identifies crucial enhancements in information integration, patient prioritization, ED provider-patient communication, and care coordination. The findings underscore key concerns about integrating advanced technologies into EDs. On the basis of these findings, this work discusses implications for designing future ED technological interventions, from enhancing communication to optimizing the integration of advanced technology and unleashing the potential of AI tools. Future efforts should further apply and test these design suggestions in real ED settings or continue using improved PD methods with functional systems that participants can interact with to further explore technological advancements in the ED diagnosis process.

## Appendix

Multimedia Appendix 1 Sample group designs by participants from remote and in-person design sessions.

## Abbreviations

AI: artificial intelligence
CDSS: clinical decision support system
ED: emergency department
EHR: electronic health record
ESI: Emergency Severity Index
PD: participatory design

## Data Availability

The datasets generated and analyzed during this study are not publicly available because the consent form for the participants stated that the data could only be accessible to the research team.
