# Supplementary material for: Designing Health Care Provider–Centered Emergency Department Interventions: Participatory Design Study
Source: JMIR Form Res. 2025 Apr 21;9:e68891. doi: 10.2196/68891 (PMC12053276; doi:10.2196/68891)

Appendix 1 - Sample Group Designs from Participants

1. Two samples from remote design sessions

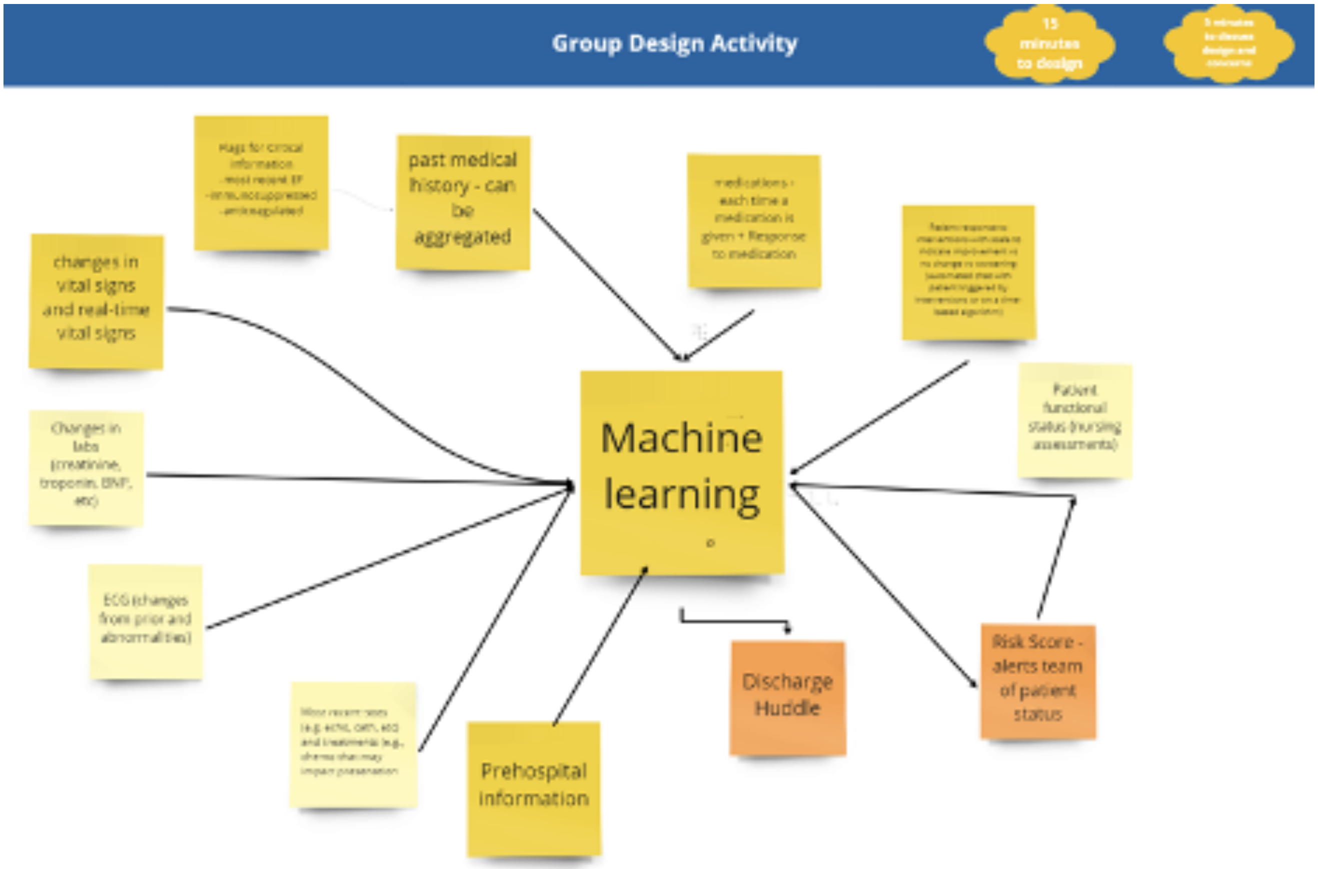

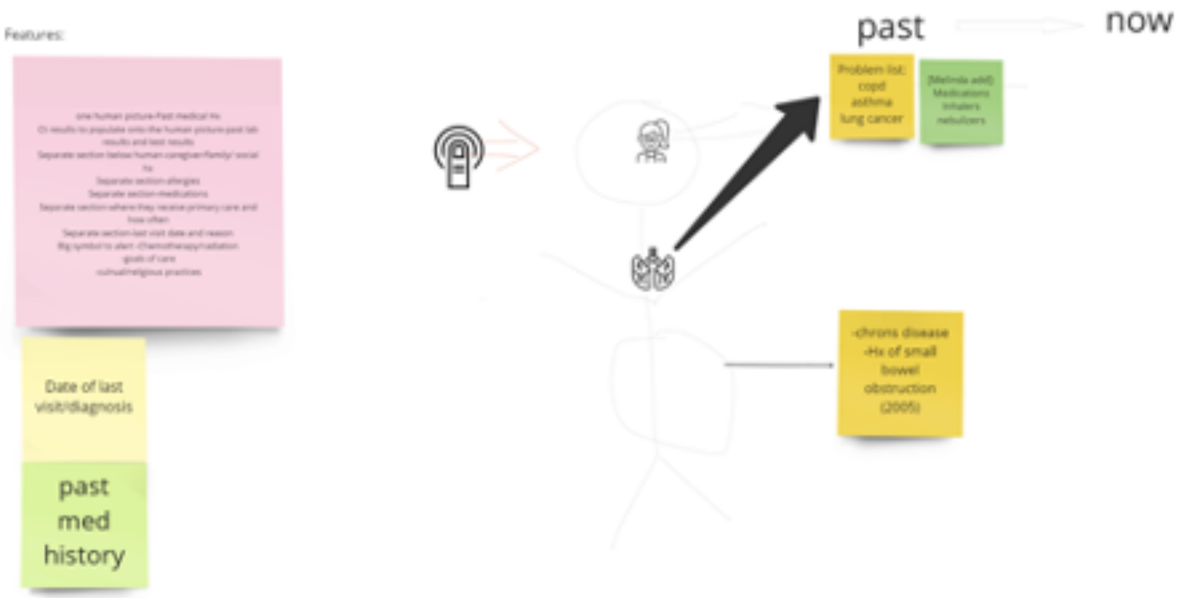

1. A sample from an in-person design session

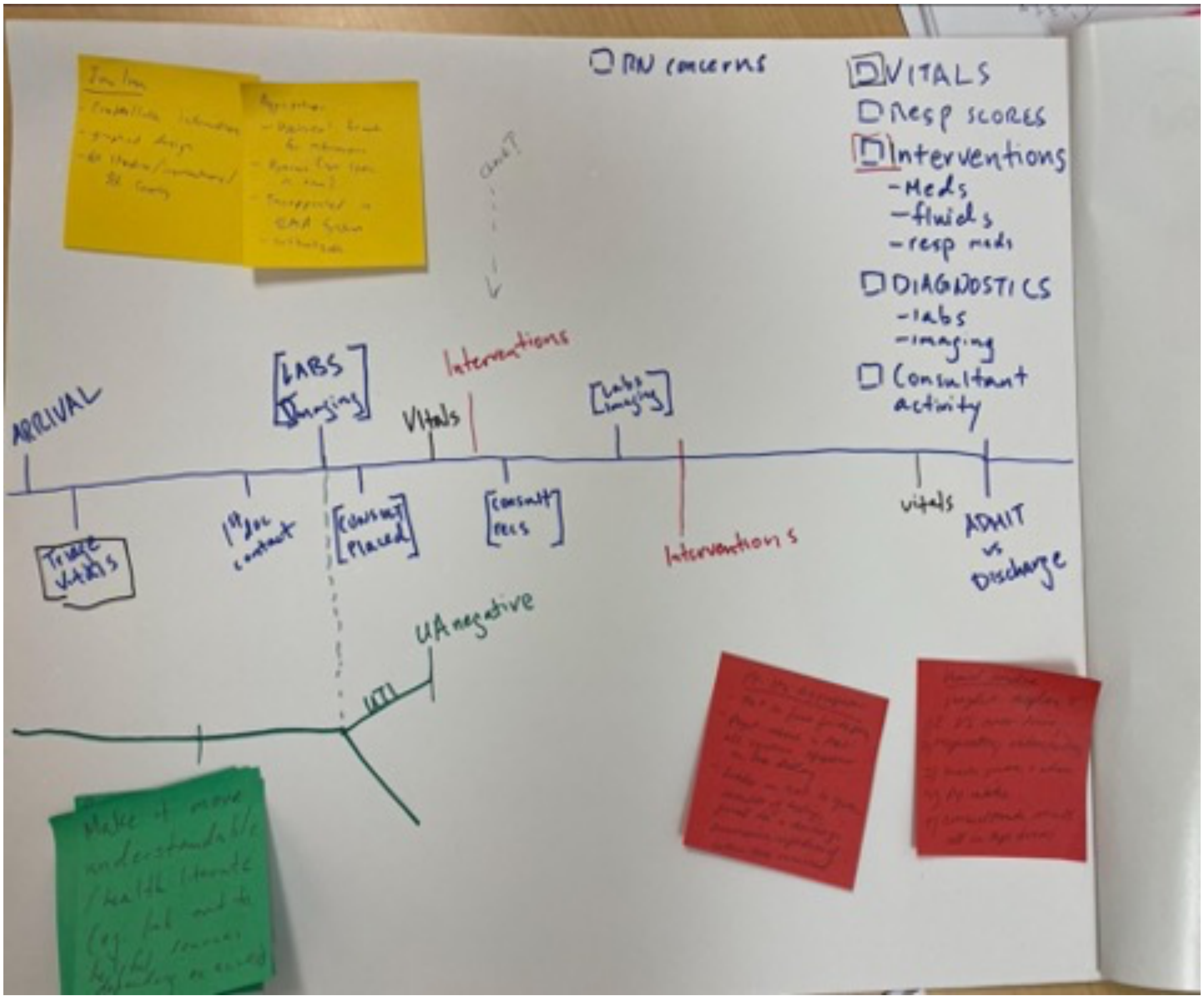

Supplement: Multimedia Appendix 1 [file formative_v9i1e68891_app1.pdf]
